# Development of *Eucalyptus* tissue culture conditions for improved *in vitro* plant health and transformability

**DOI:** 10.1186/1753-6561-5-S7-P153

**Published:** 2011-09-13

**Authors:** Cathleen Ma, Raj Deepika, Alexander A Myburg, Martin Ranik, Steven H Strauss

**Affiliations:** 1 Department of Forest Ecosystems and Society, 321 Richardson Hall, Oregon State University, Corvallis, OR, 97331-5752, USA; 2 Department of Genetics, Forestry and Agricultural Biotechnology Institute (FABI), University of Pretoria, Pretoria 0002, South Africa

## Background

Despite its importance as a widely-planted crop tree, eucalypt species and hybrids are relatively difficult to micropropagate, culture and genetically transform *in vitro*. Compared to other plant species, few non-commercial laboratories are proficient at *Eucalyptus* tissue culture and transformation. We have undertaken to establish and transform several eucalypt clones in the laboratory. Our main aims include the identification of clones amenable to culturing and transformation, and the development of robust and transferable micropropagation, organogenesis and transformation protocols to enable routine production of transgenic eucalypts for public sector research. Efficient transformation protocols are essential to take full value of the eucalypt genome for functional genomics, ecophysiology, and biotechnology.

## Methods

Five different clones of *E. grandis* (including the clone of which the genome was recently sequenced – Brasuz1) as well as a single *E. grandis* x *urophylla* hybrid were established in the laboratory. Light intensity was studied as a means to reduce browning and promote callus growth and shoot regeneration. Gibberelic acid (GA_3_) concentrations in the micropropagation medium (Murashige and Skoog’s (MS) basal medium containing 0.05 mg/L benzylaminopurine – BAP) were studied to help produce long internodes to aid transformation *in vitro*. To mitigate phenolic production we used several different antioxidants including ascorbic acid, PVP and PVPP. Shoot regeneration rates were studied by testing a concentration range of cytokinin (zeatin, benzyl aminopurine – BAP and thidiazuron - TDZ). We tested different *Agrobacterium* transformation protocol enhancements including the use of acetorsyringone and two different co-cultivation techniques (whole explant immersion versus pipetting of *Agrobacterium* suspension onto cut leaf edges). We also tested the suitability of kanamycin and hygromycin as selectable markers during transformation.

## Results and discussion

**Figure 1:**
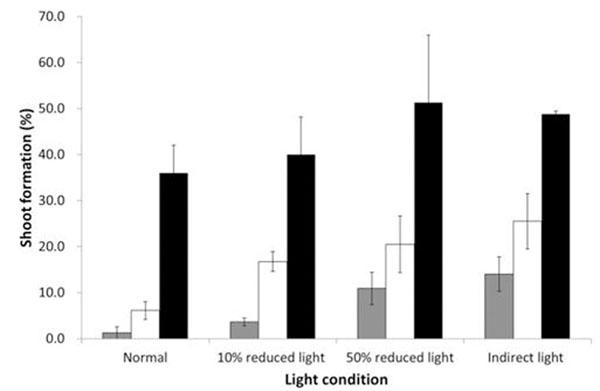
Percentage of shoot regeneration from co-cultivated leaf explants of a *E. grandic x urophylla* clone under different light conditions.

Light conditions during shoot regeneration were critical to the rate of organ regeneration, with more than 50% of explants producing shoots in the presence of reduced or indirect light, compared to approximately 30% incubated under normal light conditions. In addition, shoot differentiation occurred earlier (10-14% at 40 days) under reduced or indirect light compared to normal light (1%; Fig. 1).

**Figure 2:**
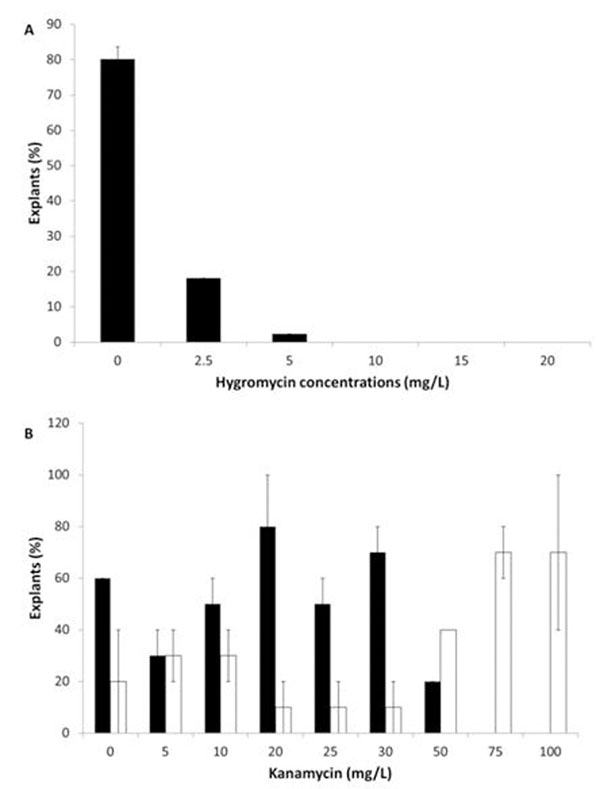
**A.** Percentage of non-transgenic explants forming callus after 30 days on shoot induction medium (SIM) with different concentrations of hygromycin. **B.** Percentage of explants forming roots (black bars) and exhibiting shoot yellowing (white bars). This experiment was conducted on a single kanamycin-resistant transgenic line of a *E. grandis x urophylla* clone after 30 days on rooting medium.

We have also begun to test the use of muslin screens to identify the best level of light exposure. The optimal level of GA_3_ in the micropropagation medium for the production of sturdy elongated internodes was found to be 0.05 mg/L. It was also apparent that the recovery of micropropagated plantlets on medium without GA_3_ for 21 days prior to internode harvesting improved plant health and produced better quality stem sections for transformation. Ascorbic acid, used as a means of reduction of phenolic browning, proved to be ineffective when used alone, even though it produced no adverse effects on callus health. By comparing several shoot induction media (SIM) for shoot regeneration from stem and leaf explants of the *E. grandis* x *urophylla* containing different cytokinin combinations, we found that the highest rates of shoot regeneration (11.8%) occurred when using stem explants incubated on SIM containing 1 mg/L NAA (nicotinamide) and 10 mg/L zeatin. Of the five tested *E. grandis* clones, we found that the highest rates of shoot regeneration (52%) were from leaf explants of clone p207 in the presence of SIM containing 3 µM TDZ and 0.1 µM NAA. Neither the use of two different acetosyringone concentrations (250 and 750 µM) during co-cultivation, nor either of the two co-cultivation techniques (immersion and pipetting) had a significant effect on transient gene expression measured shortly after cocultivation. Testing of different kanamycin and hygromycin concentrations (“kill curve”), revealed that 30 mg/L kanamycin or 5 mg/L hygromycin can be used for the selection of putative transgenics after transformation (Fig. 2).

## Conclusions

There is significant variability in the performance and response of different clones of *Eucalyptus* during micropropagation, organogenesis and transformation. Testing of multiple *Eucalyptus* clones confirmed that the genus is relatively unwieldy in tissue culture compared, for example, to *Populus.* We found that explant type (stem vs leaf for example), and quality (age, general health, absence callus browning) as well as finely-tuned phytohormone concentrations, will play critical roles in enhancing the probability of the successful generation of stable transgenic lines.

